# Tetrandrine, an Activator of Autophagy, Induces Autophagic Cell Death via PKC-α Inhibition and mTOR-Dependent Mechanisms

**DOI:** 10.3389/fphar.2017.00351

**Published:** 2017-06-08

**Authors:** Vincent Kam Wai Wong, Wu Zeng, Juan Chen, Xiao Jun Yao, Elaine Lai Han Leung, Qian Qian Wang, Pauline Chiu, Ben C. B. Ko, Betty Yuen Kwan Law

**Affiliations:** ^1^State Key Laboratory of Quality Research in Chinese Medicine, Macau University of Science and TechnologyMacau, China; ^2^Key Laboratory of Molecular Biology on Infectious Diseases, Ministry of Education, The Second Affiliated Hospital of Chongqing Medical University, Chongqing Medical UniversityChongqing, China; ^3^Department of Chemistry and State Key Laboratory of Synthetic Chemistry, University of Hong KongHong Kong, China; ^4^Department of Applied Biology and Chemical Technology, and State Key Laboratory of Chirosciences, The Hong Kong Polytechnic UniversityHong Kong, China

**Keywords:** autophagy, tetrandrine, apoptosis-resistant, mTOR, PKC-α

## Abstract

Emerging evidence suggests the therapeutic role of autophagic modulators in cancer therapy. This study aims to identify novel traditional Chinese medicinal herbs as potential anti-tumor agents through autophagic induction, which finally lead to autophagy mediated-cell death in apoptosis-resistant cancer cells. Using bioactivity-guided purification, we identified tetrandrine (Tet) from herbal plant, *Radix stephaniae tetrandrae*, as an inducer of autophagy. Across a number of cancer cell lines, we found that breast cancer cells treated with tetrandrine show an increase autophagic flux and formation of autophagosomes. In addition, tetrandrine induces cell death in a panel of apoptosis-resistant cell lines that are deficient for caspase 3, caspase 7, caspase 3 and 7, or Bax-Bak respectively. We also showed that tetrandrine-induced cell death is independent of necrotic cell death. Mechanistically, tetrandrine induces autophagy that depends on mTOR inactivation. Furthermore, tetrandrine induces autophagy in a calcium/calmodulin-dependent protein kinase kinase-β (CaMKK-β), 5′ AMP-activated protein kinase (AMPK) independent manner. Finally, by kinase profiling against 300 WT kinases and computational molecular docking analysis, we showed that tetrandrine is a novel PKC-α inhibitor, which lead to autophagic induction through PKC-α inactivation. This study provides detailed insights into the novel cytotoxic mechanism of an anti-tumor compound originated from the herbal plant, which may be useful in promoting autophagy mediated- cell death in cancer cell that is resistant to apoptosis.

## Introduction

Autophagy is an evolutionarily conserved mechanism by which cellular proteins and organelles are eliminated by the lysosomal degradation pathway (Levine and Klionsky, 2004). This process is constitutively active at a low basal level and is important for regulating cellular homeostasis by removal of superfluous organelles and proteins. Under nutrient deprivation, ER stress and hypoxia, autophagy is activated to generate free amino and fatty acids to facilitate mitochondrial production of ATP (Mizushima, 2007). An intact autophagic pathway is necessary for promoting longevity (Madeo et al., 2010) and defense against pathogens (Thurston et al., 2009), whereas dysfunction of autophagic pathway is associated with DNA damage (White, 2015), genomic instability (Mathew et al., 2007b), tumor development (Liang et al., 1999), and neurodegenerative disorders (Hara et al., 2006; Nah et al., 2015). Therefore, pharmacological intervention of the autophagic pathway can be a promising therapeutic strategy for a variety of pathological conditions. For example, the well-known mammalian target of rapamycin (mTOR) inhibitor rapamycin, and recently identified active autophagic herbal compounds onjisaponin B (Wu et al., 2013), neferine (Wong et al., 2015), hederagenin and α-hederin (Wu et al., 2017), can induce the mTOR-dependent autophagy with an accelerated clearance of mutant huntingtin fragments in Huntington's disease models (Ravikumar et al., 2004). Carbamazepine, an autophagy enhancer, was shown to ameliorate α1-antitrypsin deficiency disorder (Hidvegi et al., 2010). Many studies have revealed additional small molecules that can modulate autophagic activity (Sarkar et al., 2007; Zhang et al., 2007; Fleming et al., 2011). A group of natural alkaloid small-molecules, including isoliensinine, liensinine, dauricine, and cepharanthine (Law et al., 2014), were also reported for its ability in inducing cell death of apoptosis-resistant cells through autophagy. However, with the dual role of autophagy in cancer therapy (Mowers et al., 2017), activation of autophagy during chemotherapy may play a role in the development of anticancer drug resistance (Alderton, 2015), therefore, special cautions are required for regulating autophagy during cancer therapy.

Previously, using an image-based autophagy assay coupled with bioactivity-guided purification, we have identified alisol B as a novel autophagic inducer (Law et al., 2010). Using a similar strategy, here we report the identification of tetrandrine, a bis-benzylisoquinoline alkaloid isolated from the roots of *Radix stephania tetrandrae* (Schiff, 1987), as an inducer of autophagy. We present evidence that tetrandrine increased autophagic flux in several tumor cell lines, resulting in cell death. Moreover, tetrandrine was also cytotoxic to a panel of apoptosis-resistant cell lines. Furthermore, we showed that tetrandrine induces mTOR-dependent autophagy, and it is independent of CaMKK-β or AMPK signaling pathways. More importantly, we further showed that tetrandrine induce autophagy via direct inhibition of PKC-α. Together, our work provides novel insights into the molecular mechanism of tetrandrine-induced cell death, and identified PKC-α as a potential cellular target of tetrandrine.

## Materials and methods

### Chemicals, plasmids, small interfering RNAs, and antibodies

All chemicals were obtained from Sigma-Aldrich (St. Louis, MO) unless otherwise specified. Compound C, BAPTA/AM, E64D, pepstatin A and STO-609 were purchased from Calbiochem (San Diego, CA). Actinomycin D was obtained from Gibco® (Grand Island, NY). Iso-tetrandrine was purchased from Wako Pure Chemical Industries, Ltd (Japan). Tetrandrine (Tet) was from Sigma-Aldrich (St. Louis, MO). Fangchinoline was from Chengdu MUST Bio-technology Co. Ltd. (China). Antibodies against p70S6 kinase, phospho-p70S6 kinase (Thr389), AMPKα, phospho-AMPKα (Thr172) were purchased from Cell Signaling Technologies Inc. (Beverly, MA). p62 (SQSTM1) antibodies were purchased from Santa Cruz Biotechnology (Santa Cruz, CA). Anti-LC3 antibody was from Novus Biologicals (Littleton, CO). Anti-β-actin antibody was from Sigma. pEGFP-LC3 reporter plasmid was a gift from Prof. Tamotsu Yoshimori (Osaka University, Japan). Small interfering RNAs and non-targeting control were obtained from Qiagen (Valencia, CA).

### Cell culture

HeLa (ATCC® CCL-2™) and MCF7 (ATCC® HTB-22™) were obtained from American Type Culture Collection (ATCC) (Rockville, MD). Immortalized wild type (WT) and ATG7-deficient mouse embryonic fibroblasts (MEF) were kindly provided by Prof. Masaaki Komatsu (Juntendo University School of Medicine, Japan). Immortalized wild type and caspase 3/7-deficient MEFs were kindly provided by Prof. Richard A. Flavell (Yale University School of Medicine, United State). Immortalized wild type and caspase 8-deficient MEFs were kindly provided by Prof. Kazuhiro Sakamaki (Kyoto University, Graduate School of Biostudies, Japan). Immortalized wild type and Bax-Bak double knockout MEFs were kindly provided by Prof. Shigeomi Shimizu (Tokyo Medical and Dental University, Medical Research Institute, Japan). All medium supplemented with 10% fetal bovine serum (FBS), 50 U/ml penicillin, and 50 μg/ml streptomycin (Invitrogen, Paisley, Scotland, UK). All cell cultures were incubated at 37°C in a 5% humidified CO_2_ incubator. Iso-tetrandrine, tetrandrine and fangchinoline were dissolved in DMSO before adding to the culture medium.

### Quantification of GFP-LC3 puncta

Cells were fixed with 4% paraformaldehyde (Sigma), permeabilized with methanol, and nuclei were stained with 4′, 6-diamidino-2-phenylindole (DAPI). The localization of GFP-LC3 was examined and captured by a Photometrics CoolSNAP HQ2 CCD camera on the Olympus IX71-Applied Precision DeltaVision restoration microscope (Applied Precision, Inc, USA). To quantify autophagy, the percentage of cells with punctate GFP-LC3 fluorescence was calculated by counting the number of the cells showing the punctate pattern of GFP-LC3 in GFP-positive cells. A minimum of 300 cells from three randomly selected fields were scored per condition per experiment.

### Cytotoxicity assays and apoptosis detection

In brief, all tested compounds were dissolved in DMSO at a final concentration of 100 mmol/L. Cell viability was measured by using 3-(4,5-dimethylthiazol-2-yl)-2,5-diphenyltetrazolium bromide (MTT) reagent (Wong et al., 2005). Firstly, cells were seeded in 96-well plates for overnight incubation, and then exposed to different concentrations of tetrandrine or other tested compounds (0–100 μM) for 48 h. Ten microliter of MTT reagent was added to each well and incubated at 37°C for 4 h. This was followed by the addition of 100 μL of solubilization buffer (10% SDS in 0.01 mol/L HCl). Absorbance at OD_570nm_ was determined from each well on the next day. The percentage of cell viability was calculated using the following formula: Cell viability (%) = Cells number (treated)/Cells number (DMSO control) × 100. Apoptosis detected by annexin V staining kit (BD Biosciences) was performed according to the manufacturer's instructions. Cells were then analyzed by FACSCalibur flow cytometer (BD Biosciences) (Wu et al., 2015).

### Transmission electron microscopy

Cells were fixed overnight with 2.5% glutaraldehyde followed by a buffer wash. Samples were post-fixed in 1% OsO_4_ and embedded in Araldite 502. Ultrathin sections were double stained with uranyl acetate and lead citrate, and analyzed by Philips CM 100 transmission electron microscope at a voltage of 80 kV (Law et al., 2010).

### Real-time PCR analysis

Total RNA was extracted using RNeasy® Mini Kit (Qiagen). First strand cDNA was synthesized using the High Capacity RNA-to-cDNA Master Mix (Applied Biosystems). To detect mRNA expression levels, qPCR was performed using primers p62: 5′-GGAGCAGATGAGGAAGATCG-3′ and 5′- GACGGGTCCACTTCTTTTGA-3′. The real time PCR reactions were performed by ViiA™ 7 Real-Time PCR System (Applied Biosystems) using SYBR Green Master Mix (Applied Biosystems). Each qPCR reaction was carried out in triplicate with gene-specific amplification confirmed by melting-curve analysis.

### SDS-PAGE and western blot

Western blot were performed as described (Rouzier et al., 2005). Total S6K and 4E-BP1 level were analyzed by separating protein samples on a 10% acrylamide gel containing 0.1% methylene bisacrylamide and 13.5% acrylamide gel with 0.36% methylene bisacrylamide, respectively as described (Balgi et al., 2009).

### Biochemical kinase profiling (WholePanelProfiler)

According to ProQinase biochemical kinase assay protocol, the kinase inhibition profile of tetrandrine was determined by measuring residual activity values at two concentrations (1 and 10 μM) in singlicate in 300 wild-type protein kinase assays conducted by ProQinase^1^ (Freiburg, Germany). In brief, the compound was dissolved as 100X stock solutions in 100% DMSO. The final DMSO concentration in all reaction cocktails (including high and low controls) was 1%. A radiometric protein kinase assay (^33^PanQinase® Activity Assay) was used for measuring the kinase activity of the selected 300 protein kinases. All kinase assays were performed in 96-well FlashPlates™ from Perkin Elmer (Boston, MA, USA) in a 50 μl reaction volume. The reaction cocktail was pipetted in 4 steps in the following order: (i) 10 μl of non-radioactive ATP solution (in H_2_O), (ii) 25 μl of assay buffer/[γ-33P]-ATP mixture, (iii) 5 μl of test sample in 10% DMSO and (iv) 10 μl of enzyme/substrate mixture. The protein kinase reaction cocktails were incubated at 30°C for 60 min and then stopped with 50 μl of 2% (v/v) H_3_PO_4_. Incorporation of ^33^Pi (counting of “cpm”) was determined with a microplate scintillation counter (Microbeta, Wallac). All protein kinase assays were performed with a BeckmanCoulter Biomek 2000/SL robotic system. The difference between high and low control of each enzyme was taken as 100% activity. The residual activity (in %) for each compound well was calculated by using the following formula: Res. Activity (%) = 100 × [(signal of compound − low control)/(high control − low control)] (Karaman et al., 2008; Lotz-Jenne et al., 2016).

### Live-cell imaging

Autophagy induction was visualized in MCF-7 cells which transiently transfected with GFP-LC3, and then placed on the microscope stage covered with a 37°C chamber in which a humidified premixed gas consisting of 5% CO_2_ and 95% air was infused. After the treatment with tetrandrine (10 μM), cells with GFP-LC3 fluorescence puncta were observed using 60X Olympus PlanApoN 1.42 oil objectives at 512 nm emission. Both Differential interference contrast (DIC) and fluorescent images were acquired at 5-min intervals using high magnification widefield epifluorescence microscopy. Images were captured as serial Z-sections of 1.0 μm interval by a Photometrics CoolSNAP HQ2 CCD camera on the Olympus IX71-Applied Precision DeltaVision restoration microscope, and the epifluorescence images were numerically deconvolved using DeltaVision algorithms (Applied Precision, Inc.).

### Molecular docking study

The 3D structure of PKC-α protein was obtained from the Protein Data Bank (PDB ID: 4RA4), and then prepared with the Protein Preparation Wizard. The structures of tetrandrine and fangchinoline were downloaded from ZINC database, and preprocessed by LigPrep under the OPLS-2005 force field (Jorgensen et al., 1996). After the corresponding lowest-energy conformation of each compound was obtained, tetrandrine and fangchinoline were docked into the binding site of PKC-α protein using the Glide module (Friesner et al., 2006) with the standard precision (SP) scoring mode. The docking grid box of tetrandrine/fangchinoline was defined according to the ligand (compound 28) in the original complex (PDB ID: 4RA4). For each system, the pose with the best docking score was chosen for further analysis. The molecular docking process was carried out with the Schrödinger 2015 software.

### Statistical analysis

The results were expressed as means ± SEM. as indicated. The difference was considered statistically significant when the *P*-value was less than 0.05. Student's *t*-test or one-way ANOVA analysis was used for comparison among different groups (Law et al., 2010; Wu et al., 2013).

## Results

### Identification of tetrandrine as an inducer of autophagy using GFP-LC3 screening assay

**Figure 1:**
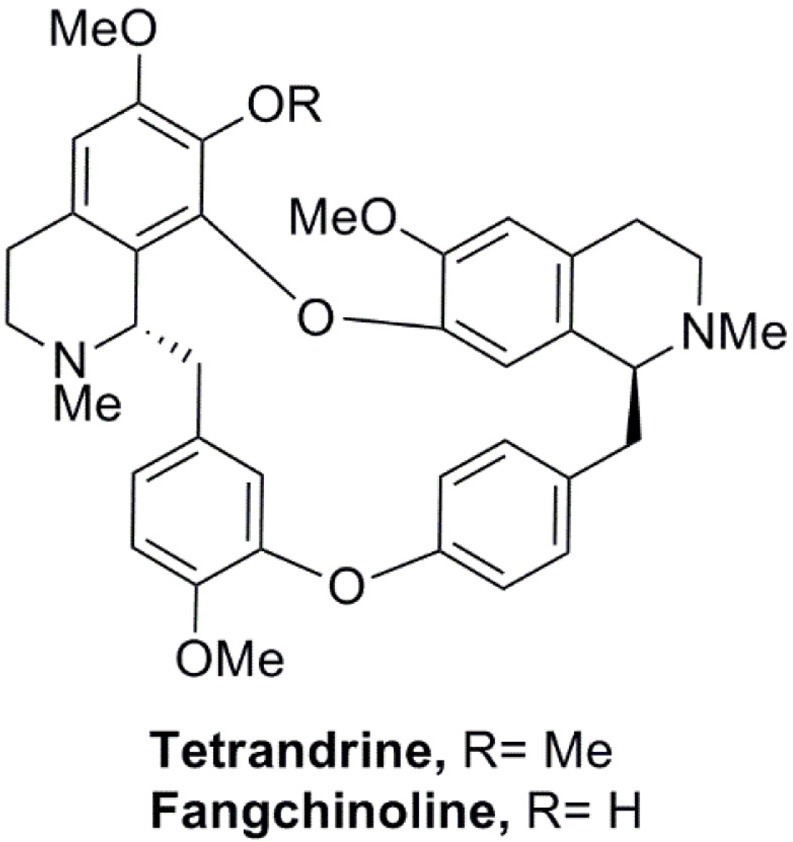
Structure of tetrandine and fangchinoline.

We have established a GFP-LC3 reporter system to screen for novel inducers of autophagy (Law et al., 2010). This was done by transient expression of GFP-LC3 reporter construct in MCF-7 cells, followed by treating cells with partially purified extracts from our natural product collection derived from traditional medicinal herbs. Cells treated with DMSO were used as negative control. Cell images in individual well were captured by fluorescence microscopy and the levels of autophagy were quantified. In the screen, among others, an ethanol-extracted fraction obtained from the root of *Stephania tetrandra* consistently increased the percentage of cells exhibiting GFP-LC3 puncta (Figure S1A). The fraction was subjected to further purification using flash column chromatography on silica gel (Figure S1B). The active compound in the purified active fraction was deduced by comparing its ^1^H and ^13^C nuclear magnetic resonance spectroscopic data (data not shown) with those of known *S. tetrandrae* components and was identified to be tetrandrine (6,6′,7,12-tetranmethoxy-2,2′-dimethyberbaman, C_38_H_42_O_6_N_2_, 622.7) (Figure 1).

### Autophagic activity of tetrandrine and its derivatives toward different cancer cell lines

**Figure 2:**
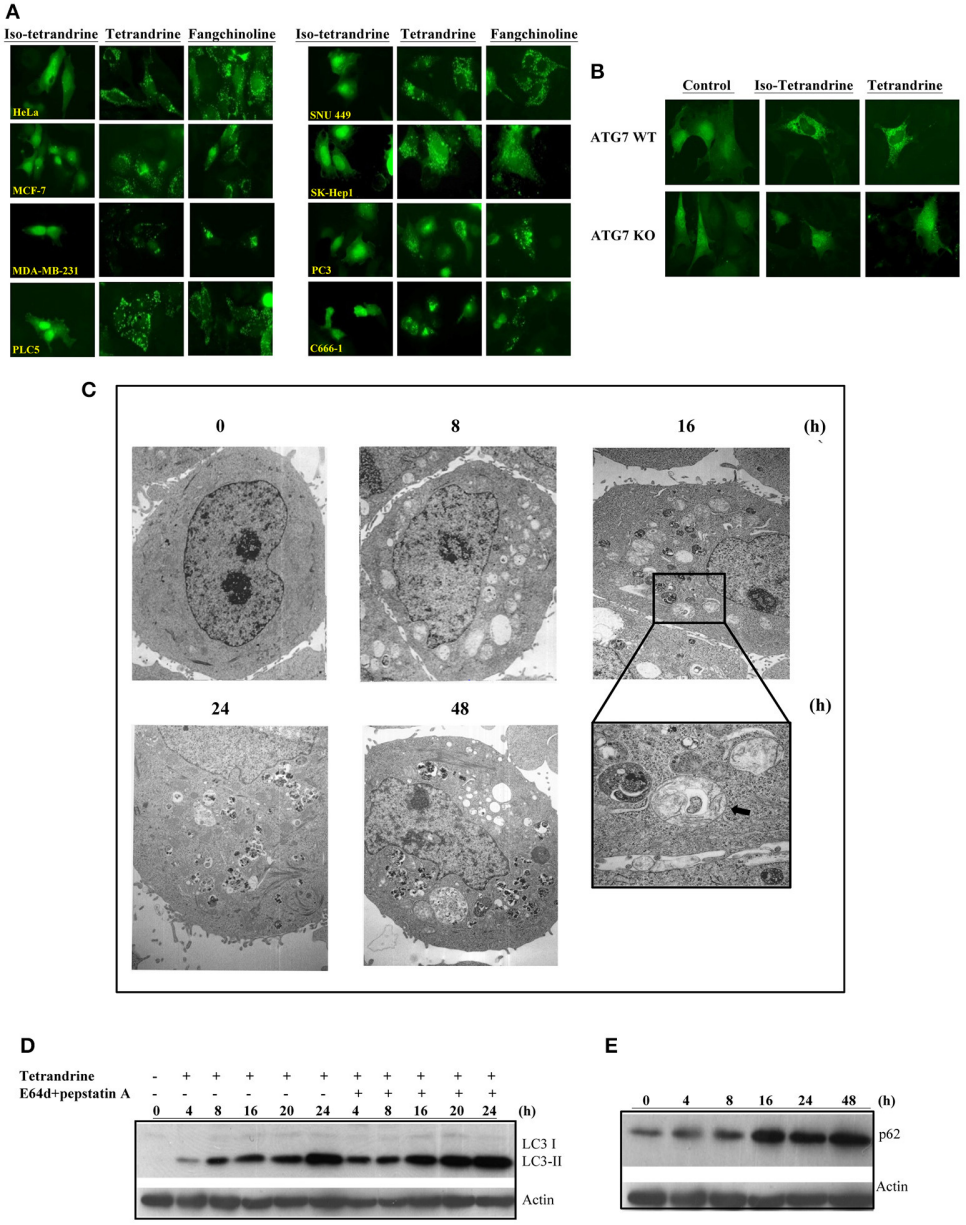
Tetrandrine (Tet) induces autophagy in multiple cell lines. **(A)** A panel of cancer cells lines expressing GFP-LC3 were treated with the indicated compounds at 5 μM for 16 h. **(B)** Both ATG7^+/+^ wild-type and ATG7^−/−^ deficient (KO) MEFs were transiently transfected with the GFP-LC3 plasmid for 24 h and then treated with DMSO (Ctrl) or 5 μM Tet for 16 h. The cells were then fixed for fluorescence imaging and cells counting. Magnification: x63. **(C)** Representative electron micrographs showing the ultrastructures of MCF-7 cells treated with Tet (5 μM) for 0–48 h. Magnification: x5600. Arrows, double-membraned autophagosomes. Magnification: x24000. **(D)** MCF-7 cells were treated with Tet (5 μM) and lysosomal protease inhibitors (E64d and pepstatin **A**) (10 μg/mL each), either alone or in combination, for 0–24 h. Cell lysates were analyzed by western blot for LC3 conversion. **(E)** Induction of p62 by Tet (5 μM) in MCF-7 cells. Cell lysates were analyzed by western blot for p62 and β-actin respectively. The results are representative of three independent experiments.

To further confirm the role of tetrandrine in the induction of autophagy, we examined the autophagic and cytotoxic activity of tetrandrine, and its structural derivatives, iso-tetrandrine (Iso-Tet) and fangchinoline respectively, using a panel of cell lines of different origins (MCF-7, PLC-5, SK-Hep1, HeLa, and PC3). Time lapse picture recording (Video 1) showed that, in MCF-7 cells, formation of GFP-LC3 puncta was observed after treatment with tetrandrine (5 μM) for 6 h (Figure S1C). Tetrandrine, and the two structurally-related derivatives, significantly induced GFP-LC3 puncta formation in all cell lines examined with similar potencies (5 μM) (Figure 2A). Tetrandrine induces GFP-LC3 puncta formation in wild-type (WT) mouse embryonic fibroblasts (MEFs), but not in Atg7-deficient MEFs (Figure 2B), suggesting that tetrandrine is a genuine autophagic inducer. Autophagic activity of tetrandrine was further confirmed by transmission electron microscopy, which revealed numerous autophagosomes in tetrandrine-treated MCF-7 cells characterized by double membrane structures, as well as autophagic vacuoles containing degraded organelles (Figure 2C). Furthermore, tetrandrine significantly increased LC3-II formation in the presence of lysosomal protease inhibitors (Figure 2D). Together these data suggested that tetrandrine is an autophagic inducer, and it induces autophagy through enhanced autophagosome formation. However, autophagic induction was associated with an unexpected upregulation of p62 (Figure 2E), which is an ubiquitin-binding protein that binds to LC3 and degraded by autophagy (Ichimura et al., 2008). While an accumulation of p62 occurs upon inhibition of autophagy (Bjorkoy et al., 2009), our subsequent analysis revealed that tetrandrine increases p62 mRNA level (Figure S2A). Accordingly, inhibition of gene transcription by actinomycin D led to a marked reduction in p62 protein level in response to tetrandrine treatment (Figure S2B), suggesting that p62 was indeed subjected to autophagic degradation.

### Tetrandrine induces autophagy through the mTOR-dependent pathway

**Figure 3:**
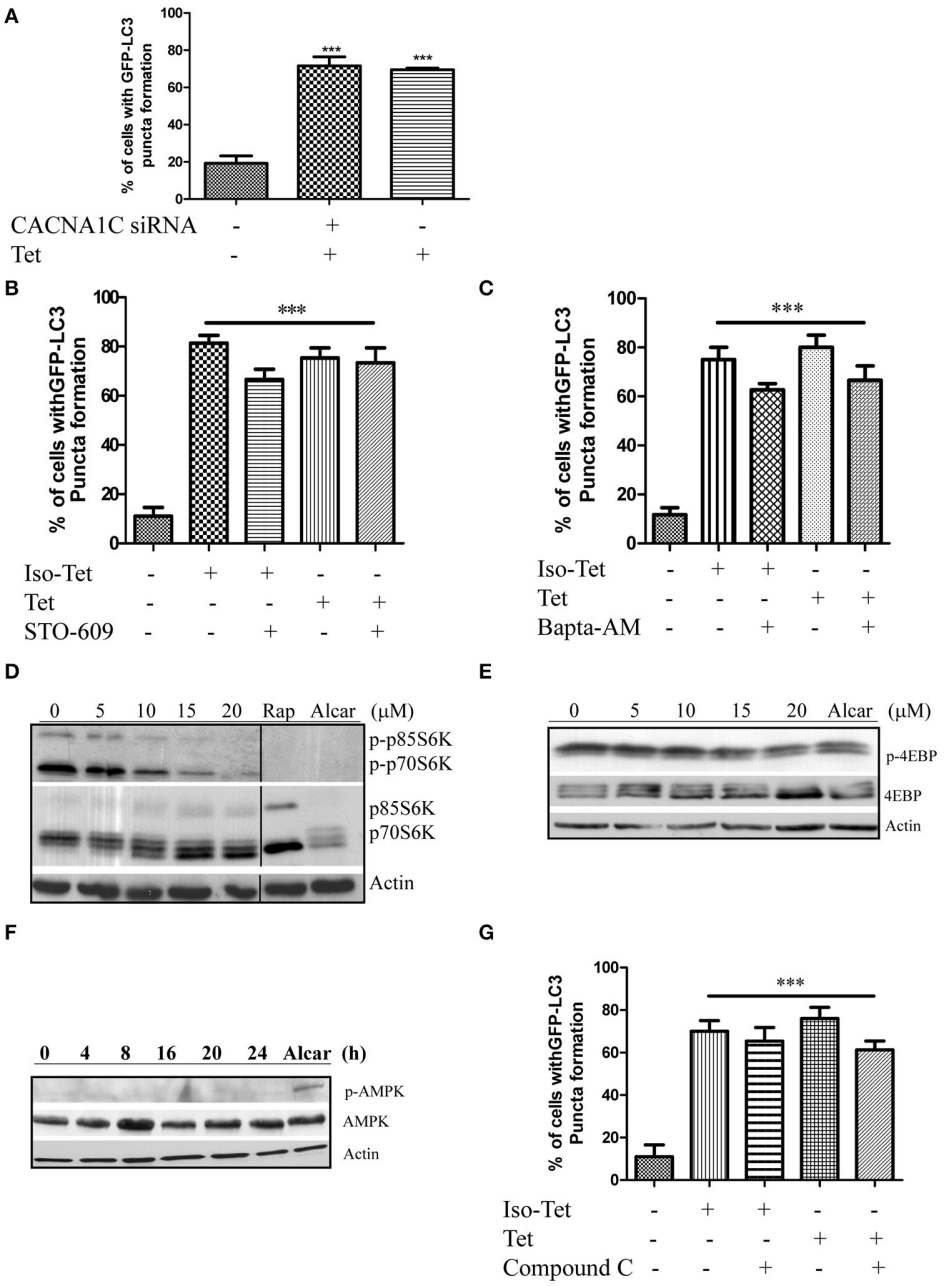
Tetrandrine induces autophagy through the mTOR-dependent pathway. **(A)** Quantitation on the percentage of CACNA1C gene knockdown MCF-7 cells with GFP-LC3 puncta formation after treatment with Tet (5 μM) for 16 h. **(B)** Bar chart showed the percentage of GFP-positive cells with GFP-LC3 puncta after 5 μM of Iso-Tet or Tet treatment in the presence or absence of STO-609 (25 μM), or **(C)** Bapta/AM (25 μM) for 16 h. **(D)** MCF-7 cells were treated with 0–20 μM of Tet for 16 h and the cell lysates were analyzed for p-p70S6K and p70S6K, **(E)** p-4EBP and 4EBP, **(F)** p-AMPK and AMPK, and β-actin respectively. Cells treated with alcar (1 mM) and rapamycin (rap) (300 nM) for 8 and 16 h respectively were used as positive control. Solid lines represent chopped image from the same gel under same exposure. **(G)** Percentage of GFP-positive cells with GFP-LC3 puncta formation after 5 μM of Iso-Tet or Tet treatment in the presence or absence of compound C (10 μM) for 16 h. Treated cells were fixed for fluorescence imaging and cells counting. Columns, means of three independent experiments; bars, SEM. ^***^p < 0.001.

Many of the signaling pathways known to regulate autophagy merge at mTOR, but emerging data also suggested that autophagy could also be induced via calcium (Ca^2+^)-associated, mTOR-independent mechanisms (Fleming et al., 2011). Tetrandrine has been suggested to act as L-type Ca^2+^ channel antagonist (Wang et al., 2004) and an inhibitor of the endosomal calcium channels (Sakurai et al., 2015). Other L-type Ca^2+^ channel antagonists, such as verapamil and loperamide (Williams et al., 2008), induces autophagy, suggesting that tetrandrine may induce autophagy via a calcium-dependent mechanism. However, depletion of CACNA1C (calcium voltage-gated channel subunit alpha1 C) of the channel in MCF-7 cells by siRNA did not attenuate tetrandrine-induced GFP-LC3 puncta formation (Figure 3A), suggesting an alternative mechanism is responsible for tetrandrine-induced autophagy. On the other hand, Ca^2+^ mobilizing agents are known to induce autophagy through the CaMKK-β-AMPK-mTOR signaling cascade (Hoyer-Hansen and Jaattela, 2007). We therefore investigated whether CaMKK-β inhibitor (STO-609) (Tokumitsu et al., 2002), or intracellular Ca^2+^ chelator (BAPTA/AM), abolishes the activity of tetrandrine on autophagy. As revealed by the quantified percentage of cells with induction of autophagy, neither STO-609 (Figure 3B) nor BAPTA/AM (Figure 3C) inhibits GFP-LC3 puncta formation induced by tetrandrine after immunocytochemistry (ICC) analysis (Figures S3A,B). Taken together, these data suggested that tetrandrine induces autophagy through a calcium-independent mechanism.

**Figure 4:**
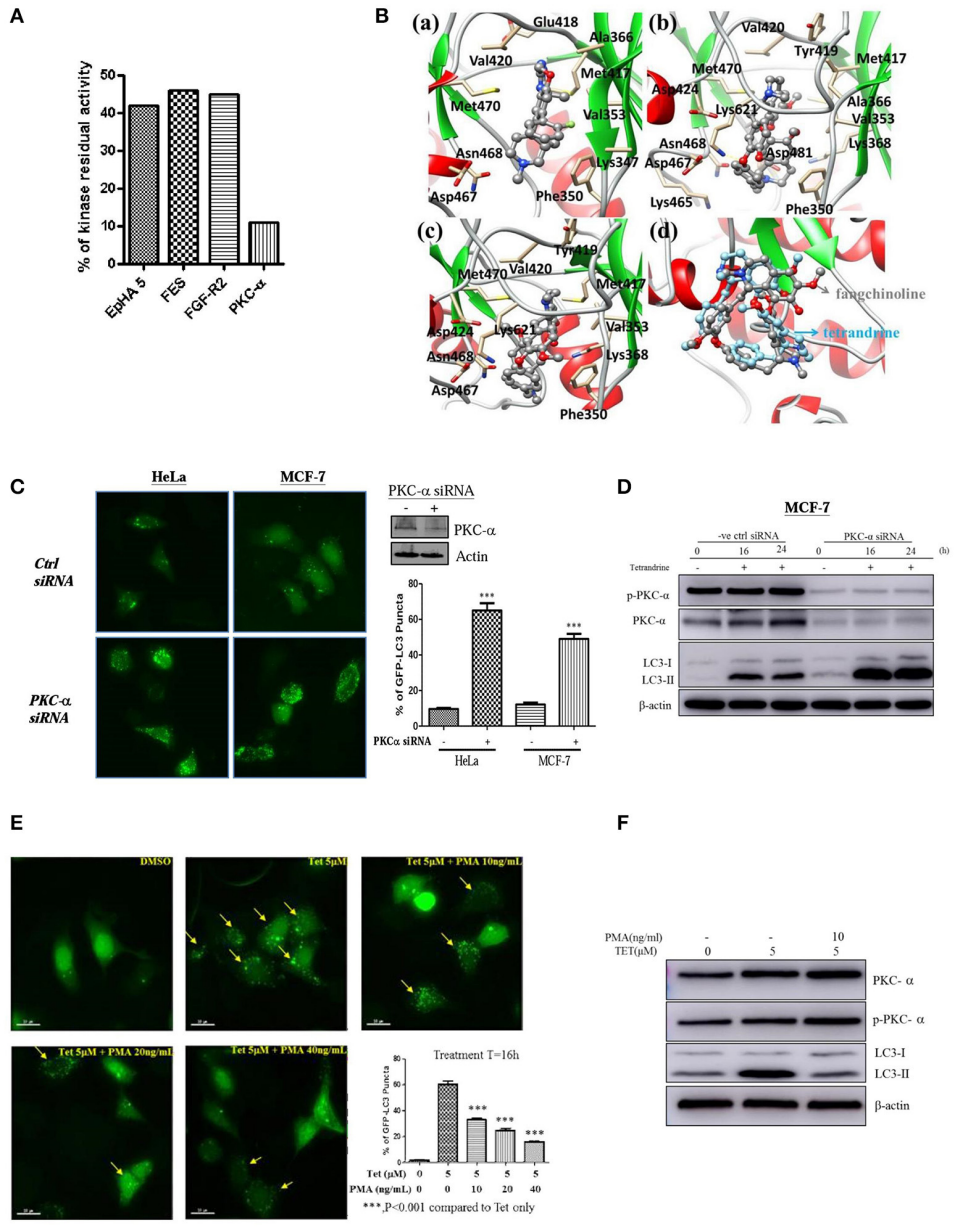
Tetrandrine induces autophagy through PKC-α inhibition. **(A)** Kinase inhibition profile of Tet was determined by measuring the residual activity values of 300 wild-type protein kinases. Bar chart represents the identification of the kinases whose residual activity dropped below 50% when treated with 1 μM of tetrandrine. Receptor tyrosine kinase (EpHA5), protein-tyrosine kinase (FES), fibroblast growth factor receptor 2 (FGFR2) and PKC-α with residual activity dropped below 50% were shown. **(B)** The binding modes of **(a)** compound 28, **(b)** tetrandrine, or **(c)** fangchinoline with PKC-α, and **(d)** the aligned conformations of tetrandrine and fangchinoline in complex with PKC-α. **(C)** HeLa and MCF-7 cells were transfected with control negative siRNA sequence or PKC-α-siRNA together with GFP-LC3 plasmid for 24 h; cells were then fixed for fluorescence microscopic analysis, and **(D)** western blot analysis for targeting PKC-α, p-PKC-α and LC3-II in MCF-7 cells, respectively. Bar chart represents the percentage of cells with GFP-LC3 puncta formation. **(E)** MCF-7 cells transiently transfected with the GFP-LC3 plasmid for 24 h were treated with DMSO (Ctrl) or 5 μM of Tet in the presence or absence of PKC-α activator, PMA, with the indicated concentrations for 16 h. Arrows indicated cells with GFP-LC3 puncta formation. Bar chart represents the percentage cells with GFP-LC3 puncta formation. Columns, means of three independent experiments; bars, SEM. ^***^p < 0.001. **(F)** MCF-7 cells were treated with DMSO (Ctrl) or 5 μM Tet in the presence or absence of PMA (10 ng/ml), for 16 h before subjecting to western blot analysis.

Autophagy acts an alternative cellular energy source under nutrient- or growth factor-deprived state by degrading intracellular materials to generate free amino and fatty acids that fuel ATP production (Mizushima, 2007). Nutrient deprivation activates autophagy through an AMPK-mTOR-dependent pathway, where activated AMPK will lead to the inactivation of mTOR (Mizushima, 2007). To elucidate the role of this pathway, we first determined if cells treated with tetrandrine resulted in the inhibition of p70S6 kinase (p70S6K) and eukaryotic translation initiation factor 4EBP, both are downstream effectors of mTOR. Cells treated with tetrandrine showed a substantial increase in the dephosphorylation of p70S6K (Figure 3D) and 4EBP (Figure 3E) respectively, suggesting the inactivation of mTOR. Nevertheless, tetrandrine treatment did not lead to the activation of AMPK (Figure 3F). Furthermore, compound C, an AMPK inhibitor, did not mitigate tetrandrine-induced GFP-LC3 puncta formation (Zhou et al., 2001; Figure 3G, Figure S3C). Together these data suggested that tetrandrine induces autophagy via an mTOR-dependent mechanism which does not involve AMPK activation.

### Tetrandrine induces autophagy via PKC-α inhibition

To further elucidate the mechanism of action of tetrandrine-induced autophagy, kinase profiling assay was conducted by screening tetrandrine against 300 recombinant kinases. The selectivity screen revealed that tetrandrine inhibits the activity of EpHA5, FES, FGF-R2, and PKC-α by over 50% respectively (Figure 4A and Table S1). Tetrandrine exhibited the highest potency on PKC-α, where there was a 89% inhibition on the enzyme activity at 1 μM, suggesting tetrandrine as a potential PKC-α inhibitor. Computational docking was used to predict the binding mode of tetrandrine in PKC-α. The molecular docking results showed that the glide score of PKC-α with tetrandrine and its analog (fangchinoline) was similar (−5.47 vs. −4.48 kcal/mol), indicating their binding motif is similar, but with relatively weaker binding affinities when compared with the reported ligand (compound 28) (−8.08 kcal/mol) in the original complex (PDB ID:4RA4). Molecular docking analysis of the compound 28 (Figure 4Ba), tetrandrine (Figure 4Bb) and fangchinoline (Figure 4Bc) revealed that all of them interact with PKC-α at similar amino acid residues, including Phe350, Val353, Met417, Val420, Met470, Asp467, and Asn468. Further structural analyses show that -COOH of Asp481 can form a strong salt bridge with tetrandrine and fangchinoline. The pi-pi stacking interaction exits between Phe350 and the phenyl group of tetrandrine or fangchinoline, further suggests that fangchinoline may adopt the similar conformation with tetrandrine when binding to PKC-α. The conformation results of fangchinoline and tetrandrine that aligned well in the complex with PKC-α (Figure 4Bd) have further confirmed tetrandrine and fangchinoline possess similar binding modes with PKC-α.

To substantiate the role of PKC-α in autophagy, we elucidated the consequence of PKC-α depletion in GFP-LC3 puncta formation. Cells expressing PKC-α siRNA but not control siRNA, exhibited a significant increase in GFP-LC3 puncta formation (Figure 4C), and LC3-II level (Figure 4D). It is noteworthy that the addition of tetrandrine further augmented LC3-II level in PKC-α knockdown cells, suggesting that tetrandrine may potentiate autophagy by inhibiting the activity of residual PKC-α. On the other hand, GFP-LC3 puncta formation (Figure 4E) and LC3-II level (Figure 4F) were suppressed by the addition of phorbol 12-myristate 13-acetate (PMA), a well-known PKC activator, in tetrandrine-treated cells. Together these data suggested that tetrandrine induces autophagy via inhibition of PKC-α.

### Tetrandrine induces autophagic cell death in apoptosis-resistant cells

**Figure 5:**
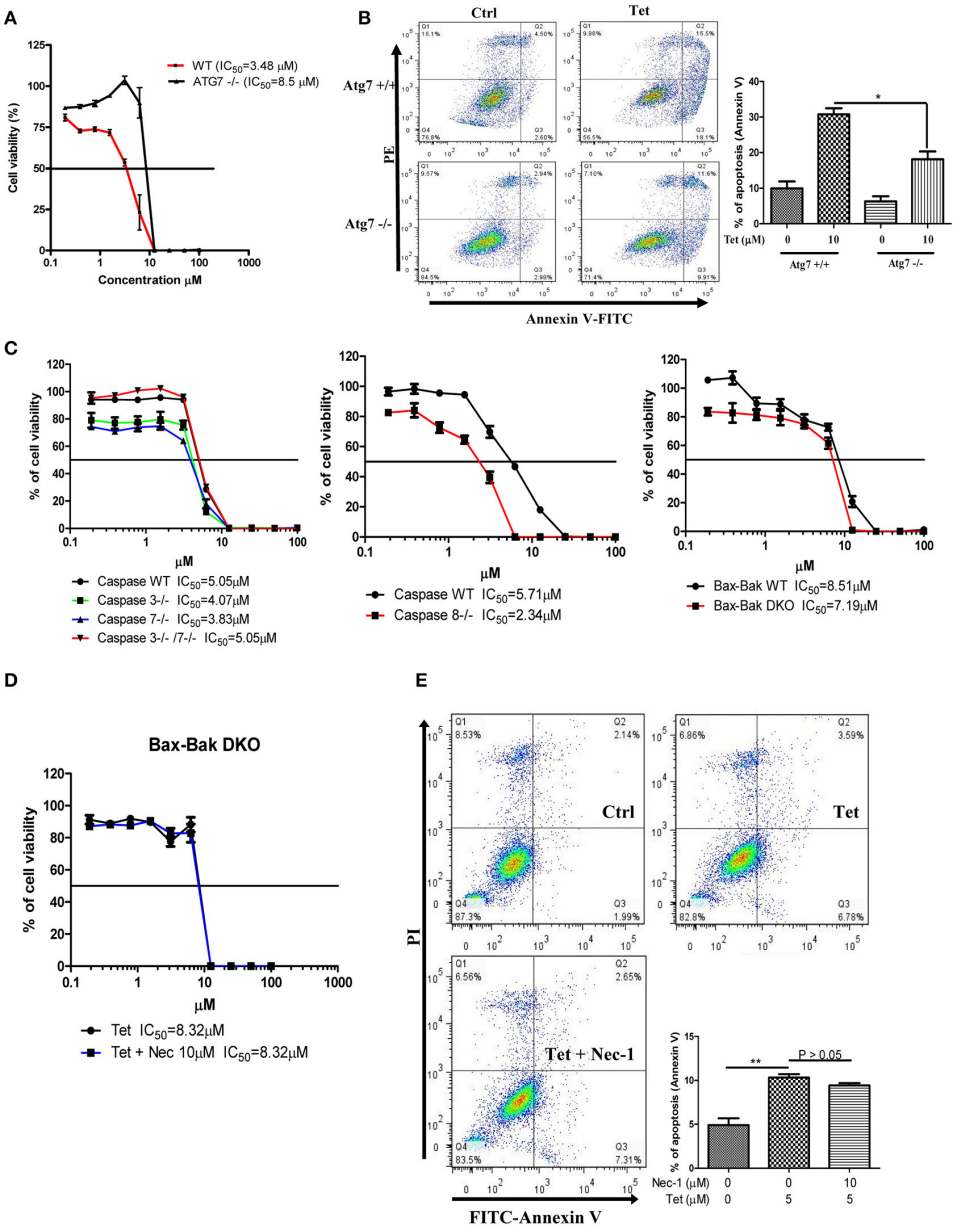
Tetrandrine induces autophagic cell death in apoptosis resistant cells. **(A)** Cytotoxicity of Tet on ATG7 wild-type and ATG7^−/−^ MEFs as measured by MTT assay, and **(B)** flow analysis after annexin V staining. **(C)** Cytotoxicity (IC_50_) of Tet on caspase 3/7 or caspase 3 and 7 knockout cells (left panel), caspase 8 knockout cells (middle panel), and Bax-Bak wild-type and DKO deficient MEFs cells (right panel). **(D)** Tet-mediated cell cytotoxicity (IC_50_) in Bax-Bak DKO MEFs with the presence of 10 μM necrostatin (Nec-1) for 24 h as measured by MTT assay, and **(E)** flow cytometry analysis after annexin V staining. Results shown are the means ± S.E.M. of three independent experiments. ^**^p < 0.01; ^*^p < 0.05.

To explore whether tetrandrine could act as an autophagic inducer with anti-cancer activity, we determined the cytotoxicity of tetrandrine, iso-tetrandrine and fangchinoline respectively, toward a panel of cancer cell lines. Tetrandrine exhibited the highest cytotoxic activity against various cell lines, whereas iso-tetrandrine and fangchinoline were less effective (Table 1). On the other hand, ATG7-deficient MEFs (IC_50_ = 8.5 μM) were more resistant to tetrandrine-induced cell death when compared to ATG-WT MEF cells (IC_50_ = 3.48 μM) (Figure 5A). This observation was further supported by the cell death analysis using annexin V staining, which demonstrated a significant higher percentage of cell death in ATG-WT MEFs when compared to ATG7-deficient MEFs after tetrandrine treatment (Figure 5B), suggesting that autophagy may be involved in tetrandrine-induced cell death.

**Table 1:** Cytotoxicity of tetrandrine and related compounds against different tumors.

**Compounds**	**IC**_**50**_ **(mmol/L)**		
**Cell type**	**Iso-tetrandrine**	**Tetrandrine**	**Fangchinoline**
HeLa	29.3 ± 0.1	11.7 ± 1.1	8.3 ± 1.5
MCF-7	26.4 ± 0.9	14.0 ± 0.6	19.2 ± 6.8
PC3	17.8 ± 2.4	8.2 ± 2.9	35.5 ± 10.5
SKHep-1	24.0 ± 2.2	9.3 ± 0.6	14.0 ± 2.7
HepG2	17.5 ± 3.5	8.2 ± 1.6	4.8 ± 0.3
PLC5	20.8 ± 3.5	8.9 ± 0.3	28.1 ± 4.8

*Cell viability was measured by MTT assay at 48 h after compounds treatment. The IC_50_ (mean ± S.E.M.) was determined graphically from the survival curves*.

Cancer cells are frequently resistant to apoptosis (Holohan et al., 2013). We therefore evaluated if tetrandrine could enhance cell death in apoptosis-resistant cancer cells. In this connection, we evaluated the cytotoxicity of tetrandrine using a panel of apoptosis-defective or apoptosis-resistant cells, including MEFs that were deficient for caspase 3, caspase 7, caspase 3 and 7 (Figure 5C), and Bax-Bak (Figure 5D) respectively. Tetrandrine exhibited similar potency toward each of these cell lines compared to the WT cells, suggesting that tetrandrine might induce cell death in the absence of apoptosis. To further evaluate if tetrandrine-induced autophagic cell death is associated with necrotic cell death, where a connection between the two mechanisms has been evidenced (Nikoletopoulou et al., 2013), Bax-Bak double knockout (DKO) MEFs were treated with tetrandrine in the presence of necrostatin (Nec-1), a widely used inhibitor of RIPK1 kinase activity which can block necrotic cell death (Vandenabeele et al., 2013). As showed in Figure 5E, Nec-1 failed to suppress cell death induced by tetrandrine, suggesting that necrotic cell death might not be involved, and further suggests the involvement of autophagy in tetrandrine-induced cell death.

## Discussion

Autophagy is a conserved cellular lysosomal degradation process responsible for the digestion and recycle of cytoplasmic materials for maintaining normal cellular homeostasis (Levine and Klionsky, 2004; Mizushima, 2007). Malfunction in autophagic pathways are frequently found in cancer (Sui et al., 2013), neurodegeneration diseases (Nixon, 2013) or myopathies (Sandri et al., 2013). With its regulatory role in the pathogenesis of cancer, loss of the autophagy gene such as beclin1 was reported in different types of human cancers (Mathew et al., 2007a). Although clinically approved agent, rapamycin, was suggested to be a potential therapeutic anti-cancer via the induction of autophagic cell death (Mathew et al., 2007a; Yang et al., 2011), it possess side effects in affecting protein synthesis and cell proliferation (Levine and Kroemer, 2008; Pallet and Legendre, 2013). Therefore, a new direction of identifying novel autophagic enhancers from natural products or herbal medicines for therapeutic application has been highlighted (Law et al., 2016).

Tetrandrine, a bisbenzylisoquinoline alkaloid, exhibits anticancer activity (Meng et al., 2004; Chen et al., 2009; Wu et al., 2010) and is used as a calcium channel blocker to treat hypertensive and arrhythmic conditions in traditional Chinese medicine (Yu et al., 2004). Recent literature has reported the novel antiviral function of tetrandrine by inhibiting the Ebola viral infection of human macrophages *in vivo*, and with high efficacy in mice model (Sakurai et al., 2015). Besides, it can inhibit wnt/β-catenin signaling and suppresses tumor of colorectal cancer (He et al., 2011). It also induces early G1 arrest accompanied by apoptosis in human colon cancer cells model (Meng et al., 2004). Although the anti-tumor action and mechanism of tetrandrine and its derivative fangchinoline have been reported, its role in autophagy induction and its potential application as an anti-tumor agent is still under investigation. By reporting a novel pathway of tetrandrine induced autophagy through PKC-α inhibition, we showed that tetrandrine exerted its autophagic effect through an AMPK independent mechanism, which did not affect the mitochondria membrane potential nor the oxidative stress pathway in MCF-7 cells (data not shown). Recent report has suggested that tetrandrine may play a therapeutic role in hepatocellular carcinoma through autophagy mediated via reactive oxygen species (ROS)/extracellular signal-regulated kinase (Gong et al., 2012). Furthermore, while Yibin Feng (Wang et al., 2011) has reported the autophagic effect of fangchinoline, via p53/sestrin2/AMPK signaling in human hepatocellular carcinoma cells, we are the first group demonstrating the autophagic effect of tetrandrine through PKC-α inhibition and mTOR inhibition in breast cancer cells, which is independent of AMPK, ROS and sestrin2 signaling pathway. Although all these studies have supported our findings that tetrandrine may play its tumor suppression role in cancer regulation through autophagy induction, the anti-cancer properties and molecular mechanisms of tetrandrine is likely to be cell-type specific, therefore, its beneficial effects in different cell types remained to be further investigated.

Although recent report showed that palmitic acid induced-autophagy is regulated via protein kinase C-mediated signaling pathway that is independent of mTOR pathways (Tan et al., 2012), other studies have demonstrated that hypercholesterolemia was associated with hyperactive signaling upstream and downstream of both mTOR complexes, including myocardial Akt, S6K1, 4EBP1, S6, and PKC-α, leading to reduced levels of myocardial autophagy (Glazer et al., 2009). Their findings further suggested that PKC-α could be down-regulated upon mTOR inhibition. Concomitantly, tetrandrine-induced autophagy was dependent on mTOR inhibition, and its effect was associated with the dephosphorylation of p70S6K and down-regulation of PKC-α. In fact, autophagy induction was found in PKC-α knockdown cells and activation of PKC-α by PMA could markedly abrogate the tetrandrine -mediated autophagy. Taken together, our findings provide a novel insight into the role of PKC-α signaling in autophagy induction by tetrandrine in breast cancer cells. PKC-α has been long recognized as important kinase for tumor growth, proliferation, survival, differentiation and motility. Many studies have reported the role of PKC-α in enhancing proliferation and anti-apoptotic signals, however, due to its very complex and highly tissue-specific functions, PKC-α can also act as a tumor promoter or a tumor suppressor depending on the cellular context. For example, PKC-α is up-regulated in bladder, endometrial, and breast cancer, but down-regulated in colorectal tumors and malignant renal cell carcinomas. Therefore, further investigation is required for interfering PKC in cancer therapy (Garg et al., 2014).

Autophagic cell death is characterized by extensive sequestration of cytoplasm leading to cell death with the formation of autophagosomes or autolysosomes (Kroemer and Levine, 2008). Recently, a new form of autophagy-dependent cell death called autosis, which is mediated by the Na^+^, K^+^-ATPase pump with unique morphological phenotype is reported. Autosis can be triggered by autophagy-inducing peptides or starvation that finally lead to excessive autophagy induction and cell death (Liu and Levine, 2015). Therefore, the mechanistic pathway regulating the form of tetrandrine -induced cell death may worth us to further investigate in the future. Although there is still no determinant evidence to support a certain form or mechanism of autophagic cell death actually exist, it is quite supportive that constitutive autophagy could finally destroy a cell and lead to cell death (Tsujimoto and Shimizu, 2005).

Recent findings suggested that cytotoxic stimuli could activate autophagic cell death in cells that are resistant to apoptosis, for example, in cells expressing anti-apoptotic Bcl-2 or Bcl-xL, or those lacking both Bax and Bak (Shimizu et al., 2004). Natural polyphenolic compounds such as curcumin, genistein, resveratrol, rottlerin, and quercetin have been reported recently for their abilities in inducing cell death via autophagy induction (Hasima and Ozpolat, 2014). Therefore, autophagy inducing compounds co-treated with standard cancer therapies have been proposed. Our recent publications have demonstrated that small molecules-mediated autophagic cell death, may work as a potential anti-cancer therapeutic approach for sensitizing apoptosis-resistant cancer cells to cell death (Wong et al., 2013; Law et al., 2014). However, during tumor development and in cancer therapy, autophagy has been reported to have dual roles in promoting both cell survival and cell death (Rosenfeldt and Ryan, 2011). Many reports have suggested the protective role of autophagy in promoting tumor cell survival under metabolic or hypoxic stressful conditions (Degenhardt et al., 2006; Karantza-Wadsworth et al., 2007; Mathew et al., 2007b). Therefore, defining the role of autophagy in different cases is critical in cancer therapy. Since autophagic cell death is involved in various human diseases, it is important to study the molecular basis of autophagic cell death in different cell types, so that specific targeted therapeutic strategies could be developed with the novel discovery of active natural products in cancer therapy.

## Author contributions

BL and VW designed, carried out the experiments, analyzed the data and prepared the draft of manuscript. WZ and JC conducted the experiments and provided materials for biological assays. XY, EL, and QW performed the computational docking and analyzed the data. PC, BK, and BL conceived the idea, supervised all research and revised the manuscript. All authors reviewed the manuscript.

### Conflict of interest statement

The authors declare that the research was conducted in the absence of any commercial or financial relationships that could be construed as a potential conflict of interest.

## Footnotes

^1^http://www.proqinase.com

## Supplementary material

Figure S1 Bioactivity guided purification of *Stephaniae tetrandrae* crude extract. **(A)** MCF-7 cells expressing GFP-LC3 were treated with the ethanol-extracted fraction (100 μg/ml) and **(B)** partially purified fractions (50 μg/ml) from the root of *Stephania tetrandra*, for 16 h. Representative fluorescence images depicting the formation of GPF-LC3 puncta were shown. **(C)** Time lapse pictures (with time lapse video, Video 1) on the induction of autophagy in MCF-7 cells treated with 5 μM of tetrandrine (duration for 6 h) were shown. The panel of time lapse fluorescent pictures showed the progressive increase in the number of GFP-LC3 punctate formation (yellow arrows) in cells from 0 to 6 h, and finally, cell death as indicated by the burst of cells (red arrows).

Figure S2 Tetrandrine increases expression of p62 mRNA level. **(A)** MCF-7 cells were treated with tetrandrine (5 μM) from 0 to 48 h. The induction of p62 mRNA were assessed by real time-PCR and normalized against β-actin as the housekeeping gene. **(B)** MCF-7 cells were pre-treated with actinomycin D (ACD) (2.5 μg/ml) for 1 h before tetrandrine (5 μM) treatment for 16 h. Cell lysates were then analyzed for p62 and β-actin. Columns, means of three independent experiments; bars, SEM. ^**^*P* < 0.01.

Figure S3 Tetrandrine induces autophagy via a calcium or AMPK independent pathway. **(A)** Representative pictures showing punctated GFP fluorescence in GFP-LC3 expressing MCF-7 cells treated with Tet (5 μM) or DMSO (ctrl) in the presence or absence of CaMKK-β inhibitor, STO-609 (25 μM), or **(B)** calcium chelator, Bapta-AM (25 μM), or **(C)** AMPK inhibitor, compound C (10 μM) for 16 h. Cells were then fixed for fluorescence imaging and cells counting. The results were representative images from three independent experiments.

Table S1 Profiling of tetrandrine at two concentrations (1 and 10 μM) against 300 wild-type protein kinases. Kinase with residual activity <50% were highlighted in yellow.

## References

[B1] Alderton G. K. (2015). Autophagy: surviving stress in pancreatic cancer. Nat. Rev. Cancer 15, 513. 10.1038/nrc4005 26299587

[B2] Balgi A. D. Fonseca B. D. Donohue E. Tsang T. C. Lajoie P. Proud C. G.. (2009). Screen for chemical modulators of autophagy reveals novel therapeutic inhibitors of mTORC1 signaling. PLoS ONE 4:e7124. 10.1371/journal.pone.0007124 19771169 PMC2742736

[B3] Bjorkoy G. Lamark T. Pankiv S. Overvatn A. Brech A. Johansen T. (2009). Monitoring autophagic degradation of p62/SQSTM1. Methods Enzymol. 452, 181–197. 10.1016/S0076-6879(08)03612-4 19200883

[B4] Chen Y. Chen J. C. Tseng S. H. (2009). Tetrandrine suppresses tumor growth and angiogenesis of gliomas in rats. Int. J. Cancer 124, 2260–2269. 10.1002/ijc.24208 19165864

[B5] Degenhardt K. Mathew R. Beaudoin B. Bray K. Anderson D. Chen G.. (2006). Autophagy promotes tumor cell survival and restricts necrosis, inflammation, and tumorigenesis. Cancer Cell 10, 51–64. 10.1016/j.ccr.2006.06.001 16843265 PMC2857533

[B6] Fleming A. Noda T. Yoshimori T. Rubinsztein D. C. (2011). Chemical modulators of autophagy as biological probes and potential therapeutics. Nat. Chem. Biol. 7, 9–17. 10.1038/nchembio.500 21164513

[B7] Friesner R. A. Murphy R. B. Repasky M. P. Frye L. L. Greenwood J. R. Halgren T. A.. (2006). Extra precision glide: docking and scoring incorporating a model of hydrophobic enclosure for protein-ligand complexes. J. Med. Chem. 49, 6177–6196. 10.1021/jm051256o 17034125

[B8] Garg R. Benedetti L. G. Abera M. B. Wang H. Abba M. Kazanietz M. G. (2014). Protein kinase C and cancer: what we know and what we do not. Oncogene 33, 5225–5237. 10.1038/onc.2013.524 24336328 PMC4435965

[B9] Glazer H. P. Osipov R. M. Clements R. T. Sellke F. W. Bianchi C. (2009). Hypercholesterolemia is associated with hyperactive cardiac mTORC1 and mTORC2 signaling. Cell Cycle 8, 1738–1746. 10.4161/cc.8.11.8619 19395857 PMC3308015

[B10] Gong K. Chen C. Zhan Y. Chen Y. Huang Z. Li W. (2012). Autophagy-related gene 7 (ATG7) and reactive oxygen species/extracellular signal-regulated kinase regulate tetrandrine-induced autophagy in human hepatocellular carcinoma. J. Biol. Chem. 287, 35576–35588. 10.1074/jbc.M112.370585 22927446 PMC3471698

[B11] Hara T. Nakamura K. Matsui M. Yamamoto A. Nakahara Y. Suzuki-Migishima R.. (2006). Suppression of basal autophagy in neural cells causes neurodegenerative disease in mice. Nature 441, 885–889. 10.1038/nature04724 16625204

[B12] Hasima N. Ozpolat B. (2014). Regulation of autophagy by polyphenolic compounds as a potential therapeutic strategy for cancer. Cell Death Dis. 5, e1509. 10.1038/cddis.2014.467 25375374 PMC4260725

[B13] He B. C. Gao J. L. Zhang B. Q. Luo Q. Shi Q. Kim S. H.. (2011). Tetrandrine inhibits Wnt/β-catenin signaling and suppresses tumor growth of human colorectal cancer. Mol. Pharmacol. 79, 211–219. 10.1124/mol.110.068668 20978119 PMC3033706

[B14] Hidvegi T. Ewing M. Hale P. Dippold C. Beckett C. Kemp C.. (2010). An autophagy-enhancing drug promotes degradation of mutant alpha1-antitrypsin Z and reduces hepatic fibrosis. Science 329, 229–232. 10.1126/science.1190354 20522742

[B15] Holohan C. Van Schaeybroeck S. Longley D. B. Johnston P. G. (2013). Cancer drug resistance: an evolving paradigm. Nat. Rev. Cancer 13, 714–726. 10.1038/nrc3599 24060863

[B16] Hoyer-Hansen M. Jaattela M. (2007). Connecting endoplasmic reticulum stress to autophagy by unfolded protein response and calcium. Cell Death Differ. 14, 1576–1582. 10.1038/sj.cdd.4402200 17612585

[B17] Ichimura Y. Kumanomidou T. Sou Y. S. Mizushima T. Ezaki J. Ueno T.. (2008). Structural basis for sorting mechanism of p62 in selective autophagy. J. Biol. Chem. 283, 22847–22857. 10.1074/jbc.M802182200 18524774

[B18] Jorgensen W. L. Maxwell D. S. Tirado-Rives J. (1996). Development and testing of the OPLS all-atom force field on conformational energetics and properties of organic liquids. J. Am. Chem. Soc. 118, 11225–11236. 10.1021/ja9621760

[B19] Karaman M. W. Herrgard S. Treiber D. K. Gallant P. Atteridge C. E. Campbell B. T.. (2008). A quantitative analysis of kinase inhibitor selectivity. Nat. Biotechnol. 26, 127–132. 10.1038/nbt1358 18183025

[B20] Karantza-Wadsworth V. Patel S. Kravchuk O. Chen G. Mathew R. Jin S.. (2007). Autophagy mitigates metabolic stress and genome damage in mammary tumorigenesis. Genes Dev. 21, 1621–1635. 10.1101/gad.1565707 17606641 PMC1899472

[B21] Kroemer G. Levine B. (2008). Autophagic cell death: the story of a misnomer. Nat. Rev. Mol. Cell Biol. 9, 1004–1010. 10.1038/nrm2529 18971948 PMC2727358

[B22] Law B. Y. Chan W. K. Xu S. W. Wang J. R. Bai L. P. Liu L.. (2014). Natural small-molecule enhancers of autophagy induce autophagic cell death in apoptosis-defective cells. Sci. Rep. 4:5510. 10.1038/srep05510 24981420 PMC4076737

[B23] Law B. Y. Mok S. W. Wu A. G. Lam C. W. Yu M. X. Wong V. K. (2016). New potential pharmacological functions of chinese herbal medicines via regulation of autophagy. Molecules 21:359. 10.3390/molecules21030359 26999089 PMC6274228

[B24] Law B. Y. Wang M. Ma D. L. Al-Mousa F. Michelangeli F. Cheng S. H. (2010). Alisol, B., a novel inhibitor of the sarcoplasmic/endoplasmic reticulum Ca^2+^ ATPase pump, induces autophagy, endoplasmic reticulum stress, and apoptosis. Mol. Cancer Ther. 9, 718–730. 10.1158/1535-7163.MCT-09-0700 20197400

[B25] Levine B. Klionsky D. J. (2004). Development by self-digestion: molecular mechanisms and biological functions of autophagy. Dev. Cell 6, 463–477. 10.1016/S1534-5807(04)00099-1 15068787

[B26] Levine B. Kroemer G. (2008). Autophagy in the pathogenesis of disease. Cell 132, 27–42. 10.1016/j.cell.2007.12.018 18191218 PMC2696814

[B27] Liang X. H. Jackson S. Seaman M. Brown K. Kempkes B. Hibshoosh H.. (1999). Induction of autophagy and inhibition of tumorigenesis by beclin 1. Nature 402, 672–676. 10.1038/45257 10604474

[B28] Liu Y. Levine B. (2015). Autosis and autophagic cell death: the dark side of autophagy. Cell Death Differ. 22, 367–376. 10.1038/cdd.2014.143 25257169 PMC4326571

[B29] Lotz-Jenne C. Luthi U. Ackerknecht S. Lehembre F. Fink T. Stritt M.. (2016). A high-content EMT screen identifies multiple receptor tyrosine kinase inhibitors with activity on TGFβ receptor. Oncotarget 7, 25983–26002. 10.18632/oncotarget.8418 27036020 PMC5041959

[B30] Madeo F. Eisenberg T. Buttner S. Ruckenstuhl C. Kroemer G. (2010). Spermidine: a novel autophagy inducer and longevity elixir. Autophagy 6, 160–162. 10.4161/auto.6.1.10600 20110777

[B31] Mathew R. Karantza-Wadsworth V. White E. (2007a). Role of autophagy in cancer. Nat. Rev. Cancer 7, 961–967. 10.1038/nrc2254 17972889 PMC2866167

[B32] Mathew R. Kongara S. Beaudoin B. Karp C. M. Bray K. Degenhardt K.. (2007b). Autophagy suppresses tumor progression by limiting chromosomal instability. Genes Dev. 21, 1367–1381. 10.1101/gad.1545107 17510285 PMC1877749

[B33] Meng L. H. Zhang H. Hayward L. Takemura H. Shao R. G. Pommier Y. (2004). Tetrandrine induces early G1 arrest in human colon carcinoma cells by down-regulating the activity and inducing the degradation of G1-S-specific cyclin-dependent kinases and by inducing p53 and p21Cip1. Cancer Res. 64, 9086–9092. 10.1158/0008-5472.CAN-04-0313 15604277

[B34] Mizushima N. (2007). Autophagy: process and function. Genes Dev. 21, 2861–2873. 10.1101/gad.1599207 18006683

[B35] Mowers E. E. Sharifi M. N. Macleod K. F. (2017). Autophagy in cancer metastasis. Oncogene 36, 1619–1630. 10.1038/onc.2016.333 27593926 PMC5337449

[B36] Nah J. Yuan J. Jung Y. K. (2015). Autophagy in neurodegenerative diseases: from mechanism to therapeutic approach. Mol. Cells 38, 381–389. 10.14348/molcells.2015.0034 25896254 PMC4443278

[B37] Nikoletopoulou V. Markaki M. Palikaras K. Tavernarakis N. (2013). Crosstalk between apoptosis, necrosis and autophagy. Biochim. Biophys. Acta 1833, 3448–3459. 10.1016/j.bbamcr.2013.06.001 23770045

[B38] Nixon R. A. (2013). The role of autophagy in neurodegenerative disease. Nat. Med. 19, 983–997. 10.1038/nm.3232 23921753

[B39] Pallet N. Legendre C. (2013). Adverse events associated with mTOR inhibitors. Expert Opin. Drug Saf. 12, 177–186. 10.1517/14740338.2013.752814 23252795

[B40] Ravikumar B. Vacher C. Berger Z. Davies J. E. Luo S. Oroz L. G.. (2004). Inhibition of mTOR induces autophagy and reduces toxicity of polyglutamine expansions in fly and mouse models of Huntington disease. Nat. Genet. 36, 585–595. 10.1038/ng1362 15146184

[B41] Rosenfeldt M. T. Ryan K. M. (2011). The multiple roles of autophagy in cancer. Carcinogenesis 32, 955–963. 10.1093/carcin/bgr031 21317301 PMC3128556

[B42] Rouzier R. Perou C. M. Symmans W. F. Ibrahim N. Cristofanilli M. Anderson K.. (2005). Breast cancer molecular subtypes respond differently to preoperative chemotherapy. Clin. Cancer Res. 11, 5678–5685. 10.1158/1078-0432.CCR-04-2421 16115903

[B43] Sakurai Y. Kolokoltsov A. A. Chen C. C. Tidwell M. W. Bauta W. E. Klugbauer N.. (2015). Ebola virus. Two-pore channels control Ebola virus host cell entry and are drug targets for disease treatment. Science 347, 995–998. 10.1126/science.1258758 25722412 PMC4550587

[B44] Sandri M. Coletto L. Grumati P. Bonaldo P. (2013). Misregulation of autophagy and protein degradation systems in myopathies and muscular dystrophies. J. Cell Sci. 126(Pt 23), 5325–5333. 10.1242/jcs.114041 24293330

[B45] Sarkar S. Davies J. E. Huang Z. Tunnacliffe A. Rubinsztein D. C. (2007). Trehalose, a novel mTOR-independent autophagy enhancer, accelerates the clearance of mutant huntingtin and alpha-synuclein. J. Biol. Chem. 282, 5641–5652. 10.1074/jbc.M609532200 17182613

[B46] Schiff P. L. Jr. (1987). Bisbenzylisoquinoline alkaloids. J. Nat. Prod. 50, 529–599. 10.1021/np50052a001 3323421

[B47] Shimizu S. Kanaseki T. Mizushima N. Mizuta T. Arakawa-Kobayashi S. Thompson C. B.. (2004). Role of Bcl-2 family proteins in a non-apoptotic programmed cell death dependent on autophagy genes. Nat. Cell Biol. 6, 1221–1228. 10.1038/ncb1192 15558033

[B48] Sui X. Chen R. Wang Z. Huang Z. Kong N. Zhang M.. (2013). Autophagy and chemotherapy resistance: a promising therapeutic target for cancer treatment. Cell Death Dis. 4, e838. 10.1038/cddis.2013.350 24113172 PMC3824660

[B49] Tan S. H. Shui G. Zhou J. Li J. J. Bay B. H. Wenk M. R.. (2012). Induction of autophagy by palmitic acid via protein kinase C-mediated signaling pathway independent of mTOR (mammalian target of rapamycin). J. Biol. Chem. 287, 14364–14376. 10.1074/jbc.M111.294157 22408252 PMC3340233

[B50] Thurston T. L. Ryzhakov G. Bloor S. von Muhlinen N. Randow F. (2009). The TBK1 adaptor and autophagy receptor NDP52 restricts the proliferation of ubiquitin-coated bacteria. Nat. Immunol. 10, 1215–1221. 10.1038/ni.1800 19820708

[B51] Tokumitsu H. Inuzuka H. Ishikawa Y. Ikeda M. Saji I. Kobayashi R. (2002). STO-609, a specific inhibitor of the Ca^2+^/calmodulin-dependent protein kinase kinase. J. Biol. Chem. 277, 15813–15818. 10.1074/jbc.M201075200 11867640

[B52] Tsujimoto Y. Shimizu S. (2005). Another way to die: autophagic programmed cell death. Cell Death Differ. 12(Suppl. 2), 1528–1534. 10.1038/sj.cdd.4401777 16247500

[B53] Vandenabeele P. Grootjans S. Callewaert N. Takahashi N. (2013). Necrostatin-1 blocks both RIPK1 and IDO: consequences for the study of cell death in experimental disease models. Cell Death Differ. 20, 185–187. 10.1038/cdd.2012.151 23197293 PMC3554339

[B54] Wang G. Lemos J. R. Iadecola C. (2004). Herbal alkaloid tetrandrine: fron an ion channel blocker to inhibitor of tumor proliferation. Trends Pharmacol. Sci. 25, 120–123. 10.1016/j.tips.2004.01.009 15058281

[B55] Wang N. Pan W. Zhu M. Zhang M. Hao X. Liang G.. (2011). Fangchinoline induces autophagic cell death via p53/sestrin2/AMPK signalling in human hepatocellular carcinoma cells. Br. J. Pharmacol. 164, 731–742. 731–742. 10.1111/j.1476-5381.2011.01349.x 21418191 PMC3188903

[B56] White E. (2015). The role for autophagy in cancer. J. Clin. Invest. 125, 42–46. 10.1172/JCI73941 25654549 PMC4382247

[B57] Williams A. Sarkar S. Cuddon P. Ttofi E. K. Saiki S. Siddiqi F. H.. (2008). Novel targets for Huntington's disease in an mTOR-independent autophagy pathway. Nat. Chem. Biol. 4, 295–305. 10.1038/nchembio.79 18391949 PMC2635566

[B58] Wong V. K. Chiu P. Chung S. S. Chow L. M. Zhao Y. Z. Yang B. B.. (2005). Pseudolaric acid B, a novel microtubule-destabilizing agent that circumvents multidrug resistance phenotype and exhibits antitumor activity *in vivo*. Clin. Cancer Res. 11, 6002–6011. 10.1158/1078-0432.CCR-05-0209 16115945

[B59] Wong V. K. Li T. Law B. Y. Ma E. D. Yip N. C. Michelangeli F.. (2013). Saikosaponin-d, a novel SERCA inhibitor, induces autophagic cell death in apoptosis-defective cells. Cell Death Dis. 4, e720. 10.1038/cddis.2013.217 23846222 PMC3730398

[B60] Wong V. K. Wu A. G. Wang J. R. Liu L. Law B. Y. (2015). Neferine attenuates the protein level and toxicity of mutant huntingtin in PC-12 cells via induction of autophagy. Molecules 20, 3496–3514. 10.3390/molecules20033496 25699594 PMC6272412

[B61] Wu A. G. Wong V. K. Xu S. W. Chan W. K. Ng C. I. Liu L.. (2013). Onjisaponin B derived from Radix Polygalae enhances autophagy and accelerates the degradation of mutant alpha-synuclein and huntingtin in PC-12 cells. Int. J. Mol. Sci. 14, 22618–22641. 10.3390/ijms141122618 24248062 PMC3856081

[B62] Wu A. G. Wong V. K. Zeng W. Liu L. Law B. Y. (2015). Identification of novel autophagic Radix Polygalae fraction by cell membrane chromatography and UHPLC-(Q)TOF-MS for degradation of neurodegenerative disease proteins. Sci. Rep. 5:17199. 10.1038/srep17199 26598009 PMC4657008

[B63] Wu A. G. Zeng W. Wong V. K. Zhu Y. Z. Lo A. C. Liu L.. (2017). Hederagenin and alpha-hederin promote degradation of proteins in neurodegenerative diseases and improve motor deficits in MPTP-mice. Pharmacol. Res. 115, 25–44. 10.1016/j.phrs.2016.11.002 27838509

[B64] Wu J. M. Chen Y. Chen J. C. Lin T. Y. Tseng S. H. (2010). Tetrandrine induces apoptosis and growth suppression of colon cancer cells in mice. Cancer Lett. 287, 187–195. 10.1016/j.canlet.2009.06.009 19586712

[B65] Yang Z. J. Chee C. E. Huang S. Sinicrope F. A. (2011). The role of autophagy in cancer: therapeutic implications. Mol. Cancer Ther. 10, 1533–1541. 10.1158/1535-7163.MCT-11-0047 21878654 PMC3170456

[B66] Yu X. C. Wu S. Chen C. F. Pang K. T. Wong T. M. (2004). Antihypertensive and anti-arrhythmic effects of an extract of *Radix stephaniae tetrandra*e in the rat. J. Pharm. Pharmacol. 56, 115–122. 10.1211/0022357022458 14980008

[B67] Zhang L. Yu J. Pan H. Hu P. Hao Y. Cai W.. (2007). Small molecule regulators of autophagy identified by an image-based high-throughput screen. Proc. Natl. Acad. Sci. U.S.A. 104, 19023–19028. 10.1073/pnas.0709695104 18024584 PMC2141901

[B68] Zhou G. Myers R. Li Y. Chen Y. Shen X. Fenyk-Melody J.. (2001). Role of AMP-activated protein kinase in mechanism of metformin action. J. Clin. Invest. 108, 1167–1174. 10.1172/JCI13505 11602624 PMC209533

