# Supplementary material for: Tetrandrine, an Activator of Autophagy, Induces Autophagic Cell Death via PKC-α Inhibition and mTOR-Dependent Mechanisms
Source: Front Pharmacol. 2017 Jun 8;8:351. doi: 10.3389/fphar.2017.00351 (PMC5462963; doi:10.3389/fphar.2017.00351)
Supplement: Figure S1 — Bioactivity guided purification of Stephaniae tetrandrae crude extract. (A) MCF-7 cells expressing GFP-LC3 were treated with the ethanol-extracted fraction (100 μg/ml) and (B) partially purified fractions (50 μg/ml) from the root of Stephania tetrandra, for 16 h. Representative fluorescence images depicting the formation of GPF-LC3 puncta were shown. (C) Time lapse pictures (with time lapse video, Video 1) on the induction of autophagy in MCF-7 cells treated with 5 μM of tetrandrine (duration for 6 h) were shown. The panel of time lapse fluorescent pictures showed the progressive increase in the number of GFP-LC3 punctate formation (yellow arrows) in cells from 0 to 6 h, and finally, cell death as indicated by the burst of cells (red arrows). [file Image1.PDF]

S1A

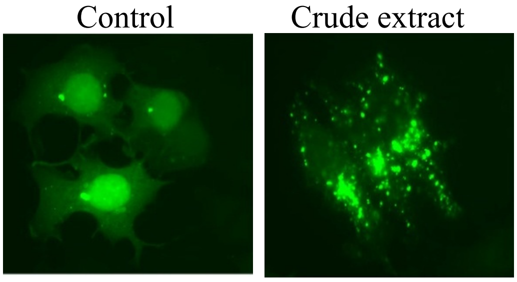

S1B

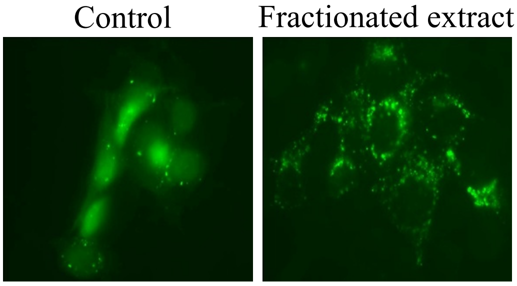

S1C

Tet 5μM; treatment duration: 6 h

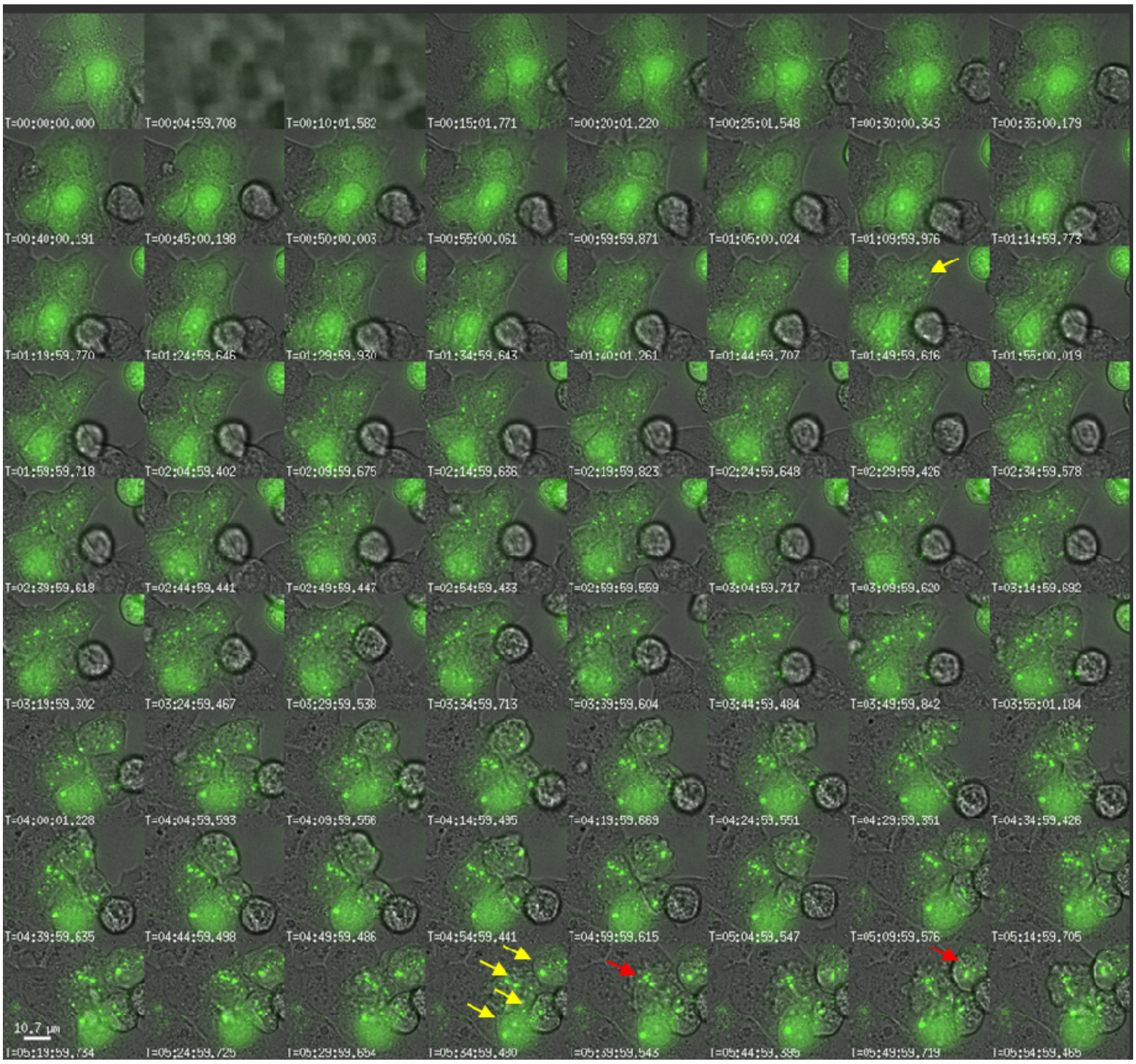

S2A

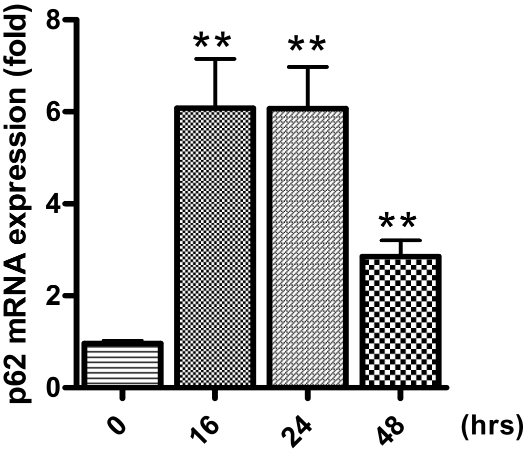
p62 mRNA expression (fold)
8
6
4
2
0
**
**
**
0
16
24
48
(hrs)

S2B

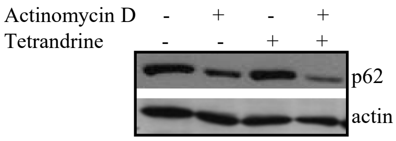
Actinomycin D - + - +
Tetrandrine - - + +
p62
actin

S3A

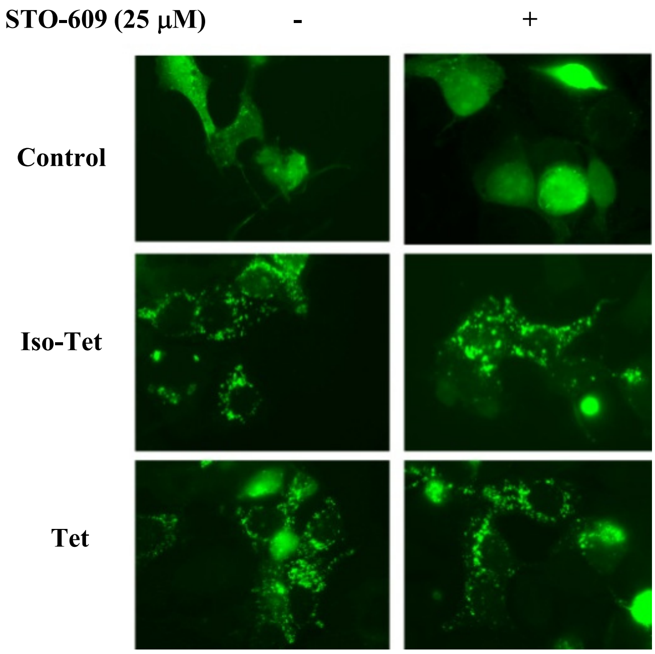
STO-609 (25 μM)
-
+
Control
Iso-Tet
Tet

S3B

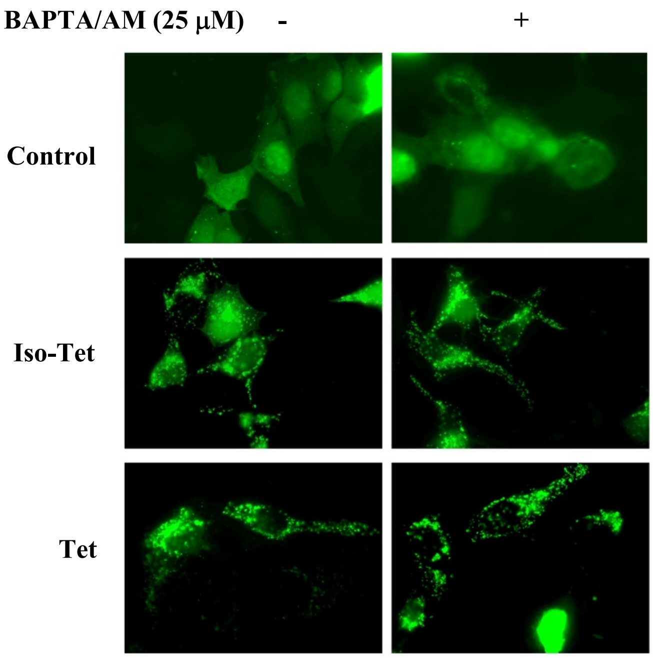
BAPTA/AM (25 μM)
-
+
Control
Iso-Tet
Tet

S3C

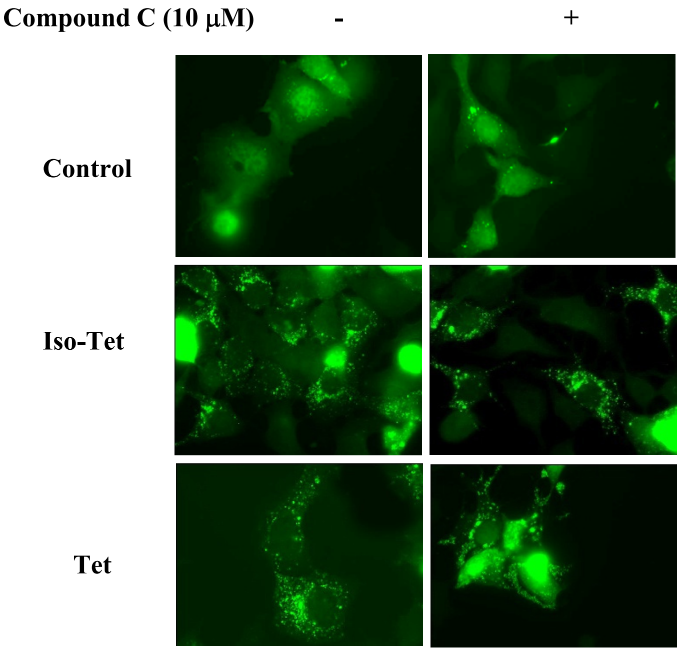
Compound C (10 μM)
-
+
Control
Iso-Tet
Tet

S4. Table

#	Kinase	1 μM	10 μM	#	Kinase	1 μM	10 μM
**1**	**ABL1**	80	77	**51**	**CHK1**	107	118
**2**	**ABL2**	84	115	**52**	**CHK2**	93	95
**3**	**ACK1**	120	91	**53**	**CK1-alpha1**	78	87
**4**	**ACV-R1**	107	100	**54**	**CK1-delta**	112	92
**5**	**ACV-R1B**	111	107	**55**	**CK1-epsilon**	85	86
**6**	**ACV-R2A**	108	109	**56**	**CK1-gamma1**	98	101
**7**	**ACV-R2B**	54	60	**57**	**CK1-gamma2**	99	106
**8**	**ACV-RL1**	95	100	**58**	**CK1-gamma3**	83	93
**9**	**AKT1**	113	103	**59**	**CK2-alpha1**	107	107
**10**	**AKT2**	96	96	**60**	**CK2-alpha2**	92	89
**11**	**AKT3**	127	114	**61**	**CLK1**	87	98
**12**	**ALK (GST-HIS-tag)**	115	97	**62**	**CLK2**	104	100
**13**	**AMPK-alpha1 aa1-550**	99	85	**63**	**CLK3**	84	90
**14**	**ARK5**	102	102	**64**	**CLK4**	109	83
**15**	**ASK1**	101	102	**65**	**COT**	97	104
**16**	**Aurora-A**	93	94	**66**	**CSF1-R**	114	84
**17**	**Aurora-B**	120	102	**67**	**CSK**	101	92
**18**	**Aurora-C**	97	84	**68**	**DAPK1**	111	101
**19**	**AXL**	92	73	**69**	**DAPK2**	131	127
**20**	**BLK**	57	83	**70**	**DAPK3**	104	102
**21**	**BMPR1A**	106	95	**71**	**DCAMKL2**	92	103
**22**	**BMX**	81	78	**72**	**DDR2**	97	88
**23**	**B-RAF**	96	100	**73**	**DMPK**	89	90
**24**	**BRK**	69	61	**74**	**DNA-PK**	95	95
**25**	**BRSK1**	97	92	**75**	**DYRK1A**	103	99
**26**	**BTK**	89	111	**76**	**DYRK1B**	107	111
**27**	**CAMK1D**	130	127	**77**	**DYRK2**	104	108
**28**	**CAMK2A**	80	55	**78**	**DYRK3**	96	82
**29**	**CAMK2B**	111	93	**79**	**DYRK4**	100	95
**30**	**CAMK2D**	104	87	**80**	**EEF2K**	125	117
**31**	**CAMK4**	78	77	**81**	**EGF-R**	72	65
**32**	**CAMKK1**	82	83	**82**	**EIF2AK2**	122	109
**33**	**CAMKK2**	92	91	**83**	**EIF2AK3**	111	105
**34**	**CDC42BPA**	102	90	**84**	**EPHA1**	77	97
**35**	**CDC42BPB**	88	92	**85**	**EPHA2**	72	69
**36**	**CDK1/CycA2**	104	97	**86**	**EPHA3**	85	73
**37**	**CDK1/CycB1**	108	95	**87**	**EPHA4**	72	81
**38**	**CDK1/CycE1**	93	98	**88**	**EPHA5**	42	38
**39**	**CDK2/CycA2**	98	95	**89**	**EPHA7**	68	63
**40**	**CDK2/CycE1**	95	93	**90**	**EPHA8**	70	71
**41**	**CDK3/CycE1**	120	99	**91**	**EPHB1**	69	60
**42**	**CDK4/CycD1**	95	87	**92**	**EPHB2**	137	122
**43**	**CDK4/CycD3**	104	91	**93**	**EPHB3**	60	76
**44**	**CDK5/p25NCK**	111	101	**94**	**EPHB4**	74	51
**45**	**CDK5/p35NCK**	106	102	**95**	**ERBB2**	99	78
**46**	**CDK6/CycD1**	78	75	**96**	**ERBB4**	74	69
**47**	**CDK7/CycH/MAT1**	108	104	**97**	**ERK1**	109	96
**48**	**CDK8/CycC**	80	104	**98**	**ERK2**	109	99
**49**	**CDK9/CycK**	93	87	**99**	**ERK7**	123	106
**50**	**CDK9/CycT1**	103	106	**100**	**FAK aa2-1052**	86	71

#	Kinase	1 μM	10 μM	#	Kinase	1 μM	10 μM
101	FER	76	65	151	MAP3K7/MAP3K7IP1	105	79
102	FES	46	55	152	MAP3K9	117	112
103	FGF-R1	93	98	153	MAP4K2	84	82
104	FGF-R2	45	56	154	MAP4K4	97	91
105	FGF-R3	112	92	155	MAP4K5	95	96
106	FGF-R4	101	88	156	MAPKAPK2	91	79
107	FGR	79	102	157	MAPKAPK3	78	87
108	FLT3	72	68	158	MAPKAPK5	97	96
109	FRK	66	68	159	MARK1	92	86
110	FYN	78	77	160	MARK2	110	115
111	GRK2	108	95	161	MARK3	83	86
112	GRK3	104	122	162	MARK4	108	105
113	GRK4	202	193	163	MATK	104	95
114	GRK5	108	113	164	MEK1	96	103
115	GRK6	109	119	165	MEK2	102	94
116	GRK7	173	167	166	MEKK2	100	104
117	GSG2	99	104	167	MEKK3	100	96
118	GSK3-alpha	96	83	168	MELK	106	89
119	GSK3-beta	106	94	169	MERTK	126	130
120	HCK	58	53	170	MET	98	97
121	HIPK1	109	102	171	MINK1	105	95
122	HIPK2	103	107	172	MKK6 S207D/T211D	91	109
123	HIPK3	104	101	173	MKNK1	110	94
124	HIPK4	104	108	174	MKNK2	95	93
125	HRI	94	89	175	MST1	85	86
126	IGF1-R	103	84	176	MST2	124	125
127	IKK-alpha	95	102	177	MST3	122	131
128	IKK-beta	119	107	178	MST4	107	112
129	IKK-epsilon	107	102	179	mTOR	73	66
130	INS-R	92	82	180	MUSK	99	92
131	INSR-R	80	93	181	MYLK	68	76
132	IRAK1	82	92	182	MYLK2	184	163
133	IRAK4 (untagged)	108	109	183	MYLK3	98	97
134	ITK	89	90	184	NEK1	105	99
135	JAK1	117	103	185	NEK11	88	79
136	JAK2	89	87	186	NEK2	111	102
137	JAK3	83	85	187	NEK3	57	57
138	JNK1	98	108	188	NEK4	78	75
139	JNK2	93	103	189	NEK6	103	110
140	JNK3	82	81	190	NEK7	113	125
141	KIT	81	65	191	NEK9	111	111
142	LCK	69	64	192	NIK	109	83
143	LIMK1	108	99	193	NLK	99	108
144	LIMK2	93	77	194	p38-alpha	101	104
145	LRRK2	93	76	195	p38-beta	98	96
146	LTK	93	97	196	p38-delta	94	92
147	LYN	52	59	197	p38-gamma	110	96
148	MAP3K1	107	107	198	PAK1	90	98
149	MAP3K10	116	95	199	PAK2	185	145
150	MAP3K11	115	96	200	PAK3	82	95

#	Kinase	1 µM	10 µM	#	Kinase	1 µM	10 µM
201	PAK4	105	95	251	S6K	98	94
202	PAK6	129	115	252	S6K-beta	113	110
203	PAK7	105	87	253	SAK	100	108
204	PASK	96	94	254	SGK1	115	102
205	PBK	125	106	255	SGK2	115	107
206	PCTAIRE1/CycY	105	110	256	SGK3	119	105
207	PDGFR-alpha	69	81	257	SLK	117	118
208	PDGFR-beta	91	81	258	SNARK	91	88
209	PDK1	98	97	259	SNF1LK2	100	109
210	PHKG1	103	100	260	SNK	95	92
211	PHKG2	99	97	261	SRC (GST-HIS-tag)	56	68
212	PIM1	85	93	262	SRMS	75	78
213	PIM2	100	97	263	SRPK1	127	110
214	PIM3	103	96	264	SRPK2	133	101
215	PKA	77	98	265	STK17A	70	79
216	PKC-alpha	11	21	266	STK23	115	124
217	PKC-beta1	97	100	267	STK25	131	150
218	PKC-beta2	88	69	268	STK33	101	110
219	PKC-delta	94	94	269	STK39	101	106
220	PKC-epsilon	64	85	270	SYK aa1-635	81	100
221	PKC-eta	99	114	271	TAOK2	110	111
222	PKC-gamma	92	88	272	TAOK3	92	91
223	PKC-iota	75	78	273	TBK1	98	97
224	PKC-mu	96	102	274	TEC	98	125
225	PKC-nu	103	100	275	TGFB-R1	91	89
226	PKC-theta	95	93	276	TGFB-R2	133	121
227	PKC-zeta	71	73	277	TIE2	88	48
228	PLK1	101	104	278	TLK1	303	329
229	PLK3	129	132	279	TLK2	306	286
230	PRK1	80	91	280	TRK-A	55	57
231	PRK2	103	105	281	TRK-B	79	58
232	PRKD2	104	141	282	TRK-C	87	70
233	PRKG1	79	83	283	TSF1	128	133
234	PRKG2	97	88	284	TSK2	109	109
235	PRKX	124	120	285	TSSK1	110	100
236	PYK2	89	78	286	TTK	104	93
237	RAF1 Y340D/Y341D (untagged)	107	103	287	TXK	93	89
238	RET	80	83	288	TYK2	90	100
239	RIPK2	97	104	289	TYRO3	85	65
240	RIPK5	125	115	290	VEGF-R1	108	84
241	ROCK1	112	122	291	VEGF-R2	111	104
242	ROCK2	123	120	292	VEGF-R3	68	65
243	RON	154	130	293	VRK1	123	116
244	ROS	97	88	294	WEE1	112	111
245	RPS6KA1	129	117	295	WNK1	66	79
246	RPS6KA2	98	94	296	WNK2	104	101
247	RPS6KA3	92	84	297	WNK3	108	116
248	RPS6KA4	97	104	298	YES	54	54
249	RPS6KA5	96	115	299	ZAK	112	101
250	RPS6KA6	106	119	300	ZAP70	69	70
